# PRLHR Immune Genes Associated With Tumor Mutation Burden can be Used as Prognostic Markers in Patients With Gliomas

**DOI:** 10.3389/fonc.2022.620190

**Published:** 2022-06-21

**Authors:** Yi Liu, Juan Xiang, Gang Peng, Chenfu Shen

**Affiliations:** ^1^ Department of Neurosurgery, Xiangya Hospital, Central South University, Changsha, China; ^2^ Department of Geriatrics, Xiangya Hospital, Central South University, Changsha, China; ^3^ National Clinical Research Center for Geriatric Disorders, Xiangya Hospital, Central South University, Changsha, China

**Keywords:** RPLHR, CIBERSORT, TMB, prognosis, glioma

## Abstract

Tumor mutation burden (TMB) is a useful biomarker for predicting the prognosis and efficacy of immune checkpoint inhibitor (ICIs). In this study, we aimed to explore the prognostic value of TMB and TMB-related PRLHR immune genes as prognostic markers in patients with gliomas. We downloaded MAF files, RNA-seq data, and clinical information from the Cancer Genome Atlas (TCGA) database. The TMB of glioma was calculated and its correlation with clinical features and prognosis was analyzed. We found that TMB was statistically correlated with the grade and age of patients with gliomas. Kaplan-Meier curve analysis showed that low TMB was associated with better clinical outcome in patients with gliomas. Additionally, a predictive model based on five HUB genes (*FABP5, VEGFA, SAA1, ADM, and PRLHR*) was constructed to predict OS in patients with gliomas. Receiver operating characteristic curve analysis shows that the model is reliable in predicting the risk of survival and prognosis. Immune microenvironment analysis revealed a correlation between TMB and infiltrating immune cells. The clinical-related immune gene, *PRLHR*, can be used as an independent prognostic factor for patients with brain glioma, and it is negatively correlated with the grade of glioma and age of patients with glioma. We found that the higher the tumor grade and the older the age, the lower the *PRLHR* expression, which was verified by CGGA database and independent experimental data. These results suggest that *PRLHR* may be a tumor suppressor for the progression of glioma and might provide a new therapeutic target for the treatment and improvement of survival rate in patients with glioma.

## Introduction

Glioma is an intractable intracranial tumor with high mortality and recurrence rate (1). Gliomas are highly invasive, making them difficult to be completely removed by neurosurgery. Traditionally, gliomas are classified into grades I to IV, including astrocytoma, oligoastrocytoma, oligodendroglioma, and glioblastoma (GBM) (2, 3). The term “low-grade glioma” (LGG) refers to grades I and II gliomas. However, LGGs include WHO grade II and III astrocytomas, oligodendrogliomas, and oligoastrocytomas. High-grade gliomas are grade III and IV gliomas (4, 5). LGG can evolve into high-grade glioma and become resistant to chemotherapy. These factors lead to the observed high mortality rate of glioma, so it is urgent to find a therapeutic target for glioma treatment (1, 6, 7).

Tumor mutation burden (TMB), or tumor mutation load, is a new biomarker of immunotherapy. TMB is defined as the number of somatic cells, codes, base substitutions, and insertion mutations per trillion bases (8). It is generally believed that TMB plays a vital role in the occurrence and development of cancer (9). In one study, cancer patients with high TMB levels showed stronger immunotherapy responses than did cancer patients with low TMB levels (10). Therefore, it is important to explore the expression of genes closely related to the TMB level. Moreover, TMB is related to the prognosis of cancer (11). Previous studies have shown that high TMB is associated with more effective immunotherapy in patients with pancreatic ductal adenocarcinoma (PDAC) and can predict patient survival (12). Results of clinical trials of immunologic drugs for non-small-cell lung cancer (NSCLC) also showed that high TMB was associated with an increase in the overall cancer response rate (ORR) and prolonged progression-free survival (PFS) (13). However, there has been less research on the role of TMB in glioma. Therefore, there is an urgent need to identify potential TMB-related gene markers to predict the prognosis of patients with gliomas.

**Figure 1:**
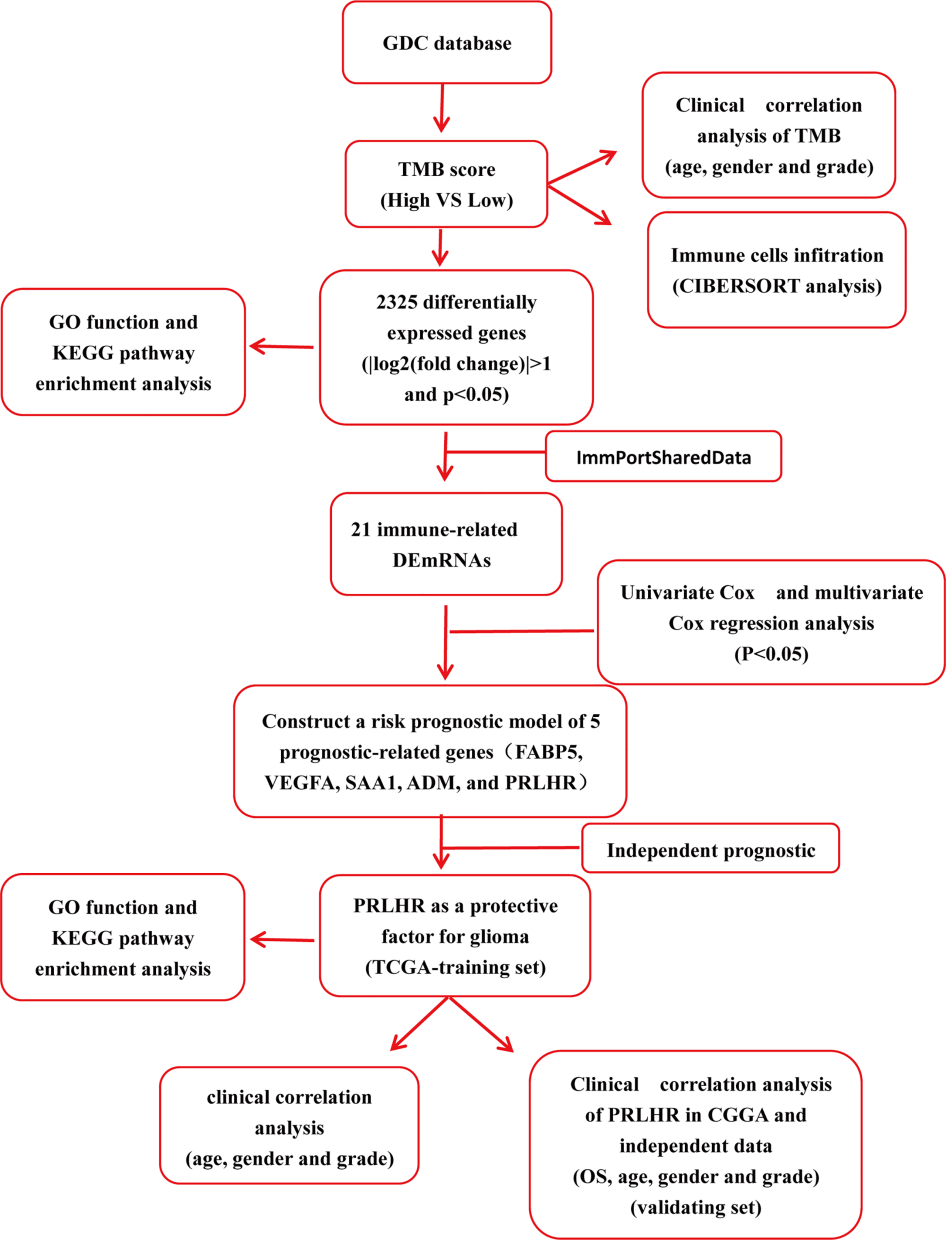
The flow chart of the study.

In this current study, we identified differentially expressed genes (DEGs) using RNA sequencing data from The Cancer Genome Atlas (TCGA) based on TMB. Then, functional enrichment analysis was further used to analyze the biological effects of these DEGs. Lastly, we identified five gene markers of glioma using the TCGA data set and bioinformatics analysis. Independent prognostic analysis showed that PRLHR could be used as an independent prognostic factor for patients with glioma, and clinical correlation analysis showed that it was significantly different from age and tumor grade. Transcriptional group data, downloaded from the Chinese Glioma Genome Atlas (CGGA) database, and external experimental data were used to verify our results. Functional enrichment analysis was used to assess the biological function of PRLHR. A flow chart of this study is shown in **Figure 1**.

## Materials and Methods

### Public Data Acquisition

The Somatic mutation data for glioma were obtained from the publicly available TCGA database through the GDC data portal (https://portal.gdc.ancer.gov/), including 393 GBM samples and 508 LGG samples. The “MaskedSomaticMutation” data set for glioma contains four types of data files; we used the “mutect” data type for the next step. The R package “maftools” (14) was used to calculate the total number of somatic cells and non-synonymous point mutations in each sample. In addition, we downloaded 703 transcriptome maps of glioma samples from TCGA, including 169 GBM samples, 529 LGG samples, and five normal brain tissues. We obtained relevant clinical information from the GDC portal, including age, sex, tumor grade, and survival outcome. mRNA data and clinical information of glioma samples were downloaded from the CGGA database (http://www.cgga.org.cn/). All data used in this part of our study are from public databases, so no informed consent was required and there are no ethical or moral conflicts to declare.

### Sample and Quantitative Real-Time Polymerase Chain Reaction Analysis

From August 2018 to May 2020, we collected frozen tissues of 71 patients who had gliomas surgically removed at the Xiangya Hospital of Central South University. RNA was extracted from cells or tissues using TRIzol Reagent (Invitrogen, CA, USA) and was reverse transcribed into cDNA using the PrimeScriptRT kit (Takara, Japan). The SYBR Green PCR MasterMix kit (Takara) and ABI7500 system (Applied Biosystems, CA, USA) for qRT-PCR. PCR amplification conditions were: initial denaturation for 30 s at 95°C, and 40 cycles at 95°C for 10 s and 60°C for 30 s. GAPDH was used as a control. The following primer sequences were used: PRLHR Forward: 5′-CCACGCCATCGACCCTTAC-3′, Reverse: 5′-CCAAGCGACCAACAGTTTGC-3′; GAPDH Forward, 5′-CCAGGTGGTCTCCTCTGA-3′ and Reverse 5′-GCTGTAGCCAAATCGTTGT-3′. PRLHR expression level was calculated by the 2−ΔΔCT method. This study was approved by the Institution Evaluation Committee of Xiangya Hospital of Central South University. All patients and their families (where appropriate) provided written informed consent to participate.

### TMB Score Calculation, Prognosis Analysis, and Clinical Correlation Analysis

TMB is defined as the total number of coding errors, base substitutions, insertions or deletions of somatic genes detected per million bases. In our study, we determined the TMB score for each sample by calculating the mutation frequency and the number of mutations/exon length (38 million). Based on the median data value, glioma samples can be divided into low and high TMB groups. We downloaded glioma clinical samples including GBM and LGG from TCGA. We excluded samples with incomplete clinical information and/or follow-up time of less than 30 days, to ensure that reliable survival time and survival status data were available, leaving 442 samples. Then, we used Perl software to merge the glioma TMB data and the corresponding survival information by searching for the appropriate sample ID. Kaplan-Meier analysis was used to compare survival differences between the low and high TMB groups, and the P value of the logarithmic rank sum test was calculated. Additionally, we evaluated the relationship between TMB levels and clinical characteristics(age, gender, and grade)using the Wilcoxon rank sum test.

### Differentially Expressed Genes

We combined the downloaded glioma transcriptional group data with sample id and TMB data, and divided glioma patients into low and high TMB groups using the median TMB value as the cut-off. The Limma software package was used to analyze the False Discovery Rate (FDR) < 0.05, | log (Folding change)| > 1) to compare TMB-related differentially genes between the two subtypes.

### Immune Cellular Infiltration Estimates

The abundance of immune cell infiltration between different populations was estimated using CIBERSORT (15). CIBERSORT is a new method widely used to characterize the cellular composition of complex tissues using the gene expression information from solid tumors (16, 17). These characteristics are highly consistent with the actual estimates of different cancers (15). When we use CIBERSORT, to assess the feasibility of leukocyte deconvolution from bulk tumors, Newman AM et al. designed and validated a leukocyte gene signature matrix, termed LM22 (15). It contains 547 genes that distinguish 22 human hematopoietic cell phenotypes, and can distinguish 22 immune cell subtypes including seven kinds of T cells (CD8+T cells, immature CD4+T cells, resting memory CD4+T cells, activated memory CD4+T cells, follicular helper T cells, regulatory T cells, and γ δ T cell), B cell types (primordial B cells, memory B cells, and plasma cells), NK cells (resting NK cells and activated NK cells), and various myeloid cells (monocytes, M0 macrophages, M1 macrophages, M2 macrophages, resting dendritic cells, activated dendritic cells, resting mast cells, activated mast cells, eosinophils, and neutrophils). In this study, the CIBERSORT online platform (http://cibersort.stanford.edu/) was used to complete the calculation, and each sample was assigned a p value. The samples with CIBERSORT output values p < 0.05 were selected for further analysis (18). The Wilcoxon rank sum test was used to compare differences abundance in immune infiltration between low group and high TMB groups. The R package “violet” is used to show the difference between the high and low TMB groups.

### Immune Related Genes

The obtained differentially expressed genes were intersected with immune genes in the ImmPortSharedData database (https://www.immport.org/) (19), and 21 immune-related differentially expressed genes were obtained (| logFC | > 2).

### Construction of Cox Proportional Hazard Model

Univariate Cox proportional hazard regression analysis and multivariate Cox regression analysis were used to analyze the differential genes related to immunity in gliomas. mRNA with P < 0.05 was screened out. On the basis of this analysis, we constructed a prognostic prediction model and obtained a comprehensive prognosis scoring system based on these differentially expressed mRNAs (DEmRNAs). The risk score was calculated as follows: Riskscore = βi × expRNAi, where expRNA is the expression level of RNA and β is the regression coefficient derived from the multivariate Cox regression model.

Based on the risk score obtained using this approach and the specific differential genes we identified, patients with glioma were divided into high risk and low risk groups. There was a difference in the overall survival (OS) rate between the two groups. The higher the risk score, the worse the prognosis of patients with glioma. Unless otherwise stated, P < 0.05 was considered statistically significant. The “Survival ROC” software package in R was used to construct 3- and 5-year time-dependent subject working characteristic (ROC) curves to evaluate the sensitivity of DEmRNAs in predicting survival. OS prediction of the DEmRNAs was analyzed by Kaplan-Meier analysis. All of these analyses were performed in R software (version 3.6.3).

### Independent Prognostic Analysis and Clinical Correlation Analysis of Prognostic Genes

To screen out independent prognostic genes in patients with glioma, we constructed a clinical model to predict survival rate. We used univariate Cox analysis and multivariate Cox regression models combined with the five prognostic genes and clinical information (including age, sex, tumor grade, and tissue type) to evaluate independent risk predictors in patients with glioma. The clinical correlation between independent prognostic genes was analyzed, and the expression of independent prognostic genes in different ages, tumor grades, and gender groups were compared. At the same time, it was verified by the CGGA database and 71 frozen specimens of glioma collected in Xiangya Hospital (independent data).

### Functional Enrichment Analysis

We combined the downloaded glioma transcriptome data with sample id and TMB data, and divided patients with glioma into low and high *PRLHR* subtypes based on median *PRLHR* expression. The Limma software package was used to compare the *PRLHR*-related differentially expressed genes between the two subtypes. Kyoto encyclopedia of genes and genomes (KEGG) and gene ontology (GO) analyses were used to evaluate the functional role of *PRLHR*-related differential genes in the prognosis of gliomas. The clusterProfiler package of R software was used for these feature rich analyses. The ggplot2 package of R software was used to display the KEGG and GO analysis results. P < 0.05 was set as the critical value.

### Statistical Analysis

All statistical analyses were performed in R software (version 3.6.3) and using GraphPad Prism 8. Unless otherwise stated, P < 0.05 was considered to be statistically significant.

## Results

### Analysis of Prognosis and the Clinical Correlation Between TMB and Patients With Glioma

**Figure 2:**
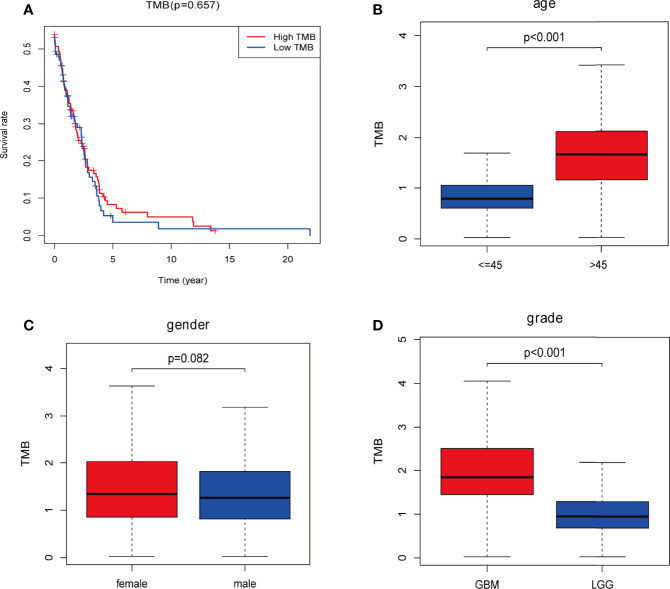
Prognosis of tumor mutation burden (TMB) and associations with risk and clinical characteristics. **(A)** Higher TMB levels correlated with poor survival outcomes with P = 5.32e-07; **(B, D)** higher TMB level is associated with age > 45 years and higher tumor grades; **(C)** no significant differences were observed for gender. Red: high-TMB, blue: low-TMB.

Downloaded 901 somatic mutation data from GDC data portal (https://portal.gdc.ancer.gov/) We determined the TMB score for each sample by calculating the mutation frequency and the number of mutations/exon length (38 million). Samples with incomplete clinical information and/or follow-up time less than 30 days were removed, which left 442 samples remaining (**Supplementary Data 1**). Kaplan-Meier analysis was used to compare survival differences between the low and high TMB groups (**Figure 2**). The OS rate of the low TMB group was significantly higher than that of the high TMB group (**Figure 2A**). Examination of the relationship between clinical information and TMB revealed a significant difference between TMB and age and tumor grade (P < 0.001), but between TMB and gender (**Figures 2B–D**). The TMB was higher in patients in the > 45 year age group than in those in the ≤ 45 year age group, suggesting that the tumor mutation load was higher and the survival prognosis was poorer in patients older than 45 years of age. TMB was higher in the GBM group than in the LGG group, suggesting that the tumor mutation load of patients with GBM is high and that their survival prognosis is poor.

### Comparison of Gene Expression Profiles Between Low and High TMB Groups

**Figure 3:**
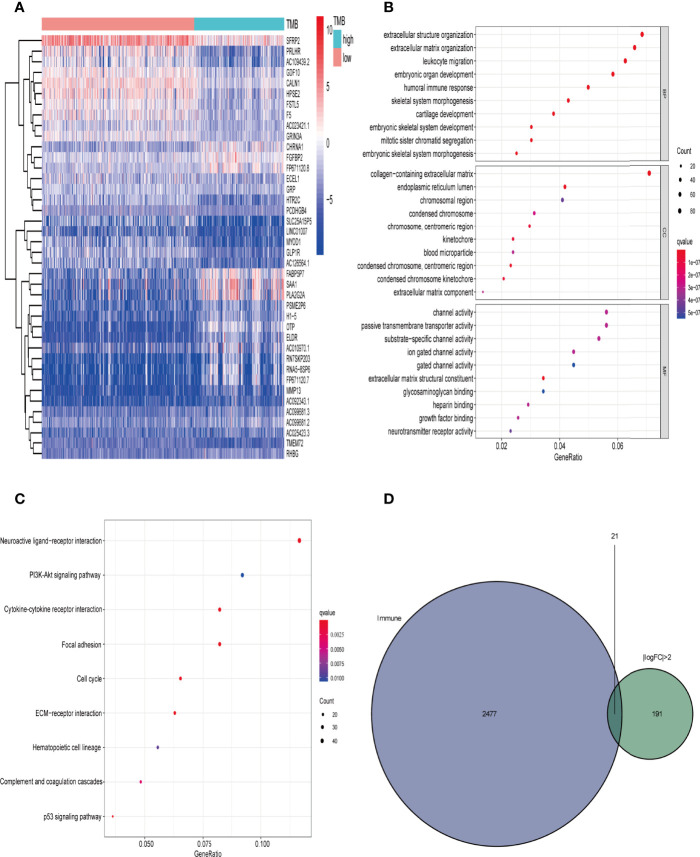
Comparison of gene expression profiles in low- and high-TMB samples. **(A)** Top 40 differentially expressed genes (DEGs) are shown in a heatmap plot (red: low-TMB, cyan: high-TMB); **(B, C)** GO (biological process [BP], cellular component [CC], and molecular function [MF]) and KEGG pathway enrichment results, respectively, revealed that these genes are involved in immune-related pathways (top 10); **(D)** identification of tumor mutation burden (TMB)-related immune-genes.

We downloaded the transcriptome map and somatic mutation data of 698 cases of gliomas from TCGA database, and obtained TMB data for 684 (low TMB group n = 429, high TMB group n = 255). We used the limma package for difference analysis, with FDR < 0.05, | log (folding change) > 1 as the screening conditions. Using this approach we identified 2325 DEGs and, consistent with the heat map results, saw that the gene expression level was generally lower in the high TMB group than in the low TMB group (**Figure 3A**).

We then performed GO enrichment and KEGG pathway analyses. GO enrichment analysis results showed that the DEGs were mainly involved in humoral immune response, leukocyte migration, circulating immunoglobulin-mediated humoral immune response, humoral immune response regulation, and other immune-related functions (**Figure 3B**). In addition, KEGG pathway analysis results included immune-related PI3K-Akt signal pathway and the tumor-related p53 signal pathway(**Figure 3C**). Since TMB is related to immune signals or immune pathways in gliomas, we further downloaded 2498 immune-related genes from the Immport database to intersect with the DEGs (| logFC | > 2), and selected 21 immune-related genes for follow-up analysis (**Figure 3D** and **Table 1**).

**Table 1:** Immune-related genes differentially expressed in low and high TMB groups.

Gene symbol	Low group	High group	logFC	P value	FDR
*IGLV3-25*	0.595280902	2.656306995	2.157779376	2.75E-07	5.20E-07
*FABP5*	4.493339423	27.16374463	2.595822423	2.71E-38	3.84E-36
*PI3*	3.61878267	17.09016606	2.239590042	5.28E-20	2.95E-19
*AREG*	0.247854173	1.015805753	2.035061096	5.13E-15	1.79E-14
*CAMP*	0.084737702	0.623194362	2.878606276	7.86E-12	2.12E-11
*IGHG4*	0.763233722	4.524695111	2.567623761	3.87E-15	1.36E-14
*SAA1*	4.901877373	38.0663075	2.957108334	1.55E-34	7.57E-33
*LTF*	16.30287517	105.648794	2.696077974	1.59E-30	3.81E-29
*TRAV36DV7*	0.099436409	0.400656279	2.010518986	1.37E-23	1.16E-22
*PLA2G2A*	1.851061719	25.36095834	3.776184359	4.64E-35	2.58E-33
*NPPB*	0.067783111	0.431452502	2.67020399	4.68E-05	7.38E-05
*TRDC*	0.942699545	6.519541447	2.789900559	8.53E-29	1.53E-27
*ADM*	3.826489992	15.40912532	2.00969144	1.71E-30	4.05E-29
*IGHD*	0.100028925	0.542879429	2.440214577	2.74E-05	4.42E-05
*PRLHR*	4.782234971	0.742983801	-2.686282355	9.70E-44	9.14E-41
*GDF10*	4.539535808	1.084341077	-2.065726157	1.40E-33	5.83E-32
*VEGFA*	5.077718004	22.2783962	2.133393197	1.73E-32	5.90E-31
*GRP*	2.468911159	0.46263386	-2.415932157	6.59E-05	0.000102575
*SAA2*	0.420412631	2.788398825	2.729559005	3.52E-24	3.24E-23
*MMP9*	3.113564813	13.0472534	2.067106918	9.31E-30	1.96E-28
*GLP1R*	0.737232225	0.119682776	-2.622903593	6.18E-27	8.21E-26

### Differential Abundance of Immune Cells in the Low and High TMB Groups

**Figure 4:**
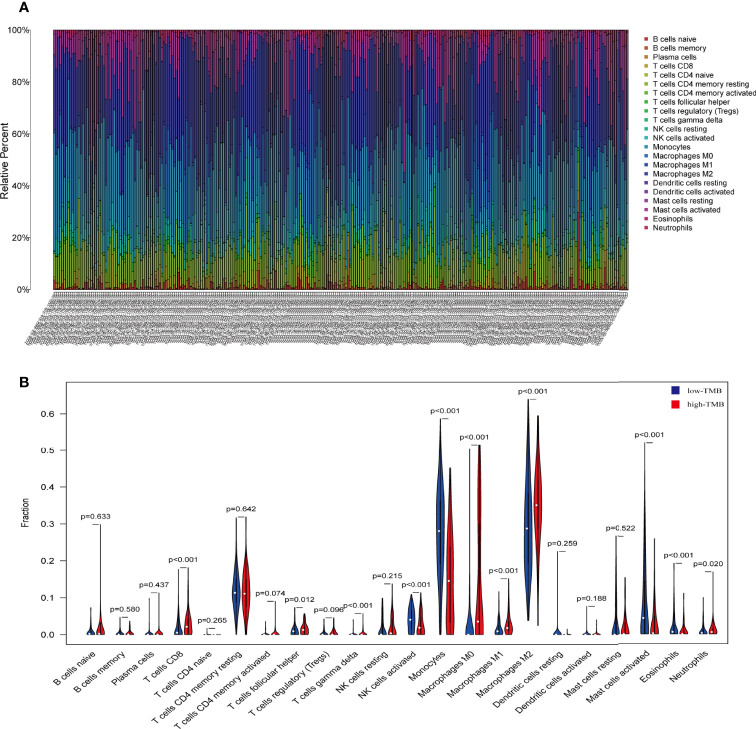
The proportion of 22 important immune cells in low- and high-tumor mutation burden (TMB) groups. **(A)** The specific 22 immune fractions represented by various colors in each sample are shown in a bar plot. **(B)** Wilcoxon rank sum test showed that the infiltration of CD8+T cells, helper T cells, γ δ T cells (T cells gamma delta), M0, M1, M2, and neutrophils was greater in the high-TMB group than in the low-TMB group. Activated NK cells, monocytes, and activated mast cells were higher in the low-TMB group than in the high-TMB group. Red: high-TMB, blue: low-TMB.

We predicted that the primary function of the DEGs is related to immunity, so we wanted to compare immune components in the high and low TMB groups. The immunocyte content of the data from 684 transcriptional groups was calculated using the CIBERSORT software package. After screening with P < 0.05, 266 immune cell content samples were obtained. These included 102 low TMB group cases and 164 high TMB group cases. The abundance of 22 different immune cells was assessed in each glioma sample (**Figure 4A**). The Wilcoxon rank sum test showed that the infiltration levels of CD8+T cells, helper T cells, γ δ T cells (T cells gamma delta), M0, M1, M2, and neutrophils in the high TMB group were higher than those in the low TMB group. The levels of activated NK cells, monocytes, and activated mast cells were higher in the low TMB group than in the high TMB group (**Figure 4B**).

### Five Immune Genes Related to the Prognosis of Gliomas

To determine the relationship between the 21 differentially expressed immune-related genes and the prognosis of patients, we screened 354 transcriptional progenitor data from 698 gliomas for further analysis. The screening criteria were the presence of complete clinical data and a follow-up time of more than 30 days (**Table 2**). Each immune gene was subjected to univariate Cox proportional hazard regression. Using this approach, we found that 15 differentially expressed immune-related genes had significant prognostic value (P < 0.05; **Table 3**). Then, prediction models were constructed based on the coefficients of five differentially expressed genes identified by multivariate Cox proportional hazard regression analysis: *FABP5, VEGFA, SAA1, ADM*, and *PRLHR* (P < 0.05; **Table 4**). The formula used to calculate the risk score was: (0.0153071550563824*exp(FABP5)) + (0.0139157402582103*exp(VEGFA)) + (0.00245933668114062*exp(SAA1)) + (-0.0183653661586793*exp(ADM)) + (-0.0996621554743428*exp (pPRLHR)).

**Table 2:** Analysis of the clinical characteristics of patients from multiple institutions.

Characteristic	TCGA n = 354	CGGA n = 278	Independent n = 71
Age, year	<=45y (183 51.69%) > 45y (171,48.31%)	<=45y (173, 62.23%) > 45y (105, 37.77%)	<=45y (33,46.48%) > 45y (38, 53.52%)
Gender Male Female	189 (53.38%) 165 (46.61%)	116 (41.72%) 162 (58.27%)	34 (47.89%) 37 (52.11%)
Grade I-II III-IV	297 (83.89%) 57 (16.1%)	104 (37.41%) 174 (62.59%)	30 (42.25%) 41 (57.75%)
OS state Alive Dead	165 (46.61%) 189 (53.38%)	98 (35.25%) 180 (64.75%)	49 (69.01%) 22 (30.99%)

**Table 3:** Univariate Cox analysis of 15 differentially expressed immune-related genes.

Gene	HR	HR.95L	HR.95H	coxPvalue
*PI3*	1.00277051565192	1.00070938690889	1.00483588963512	0.00840217130095769
*MMP9*	1.02122749891129	1.00968426177984	1.03290270435057	0.000292730264886043
*LTF*	1.00060844267178	1.00021957682276	1.00099745970425	0.00216209422991424
*FABP5*	1.01884063827592	1.01266789347514	1.02505100921122	1.74465272373071e-09
*VEGFA*	1.01124572728486	1.0048390137748	1.01769328910736	0.000563430807949492
*PLA2G2A*	1.00486128892917	1.00248020458543	1.00724802880869	6.16319158733888e-05
*SAA1*	1.00393194187812	1.00209007915377	1.00577718998506	2.80893024223731e-05
*SAA2*	1.06948724207434	1.03380613246787	1.10639986070631	0.00010429614932004
*ADM*	1.01309575802278	1.00401048188133	1.02226324669493	0.00464327452818587
*AREG*	1.15228153362982	1.00009117873219	1.32763168097079	0.0498527220218426
*GDF10*	0.914163568928172	0.853920066213302	0.978657211396111	0.00987378028301774
*NPPB*	2.36004859792531	1.14250502450986	4.8751027479802	0.0203467392385079
*PRLHR*	0.863557965961011	0.804719804318929	0.926698158256296	4.61393863957881e-05
*TRAV36DV7*	1.379826478417	1.08945823081344	1.74758522786055	0.00756932250949694
*TRDC*	1.01009889884043	1.00006235299744	1.02023617065532	0.0485865710437049

**Table 4:** Multivariate Cox analysis of 5 differentially expressed immune-related genes.

Gene	Coef	HR	P value
*FABP5*	0.015307155	1.01542491	<0.001
*VEGFA*	0.01391574	1.014013015	0.003
*SAA1*	0.002459337	1.002462363	<0.001
*ADM*	-0.018365366	0.98180225	<0.001
*PRLHR*	-0.099662155	0.905143164	<0.001

**Figure 5:**
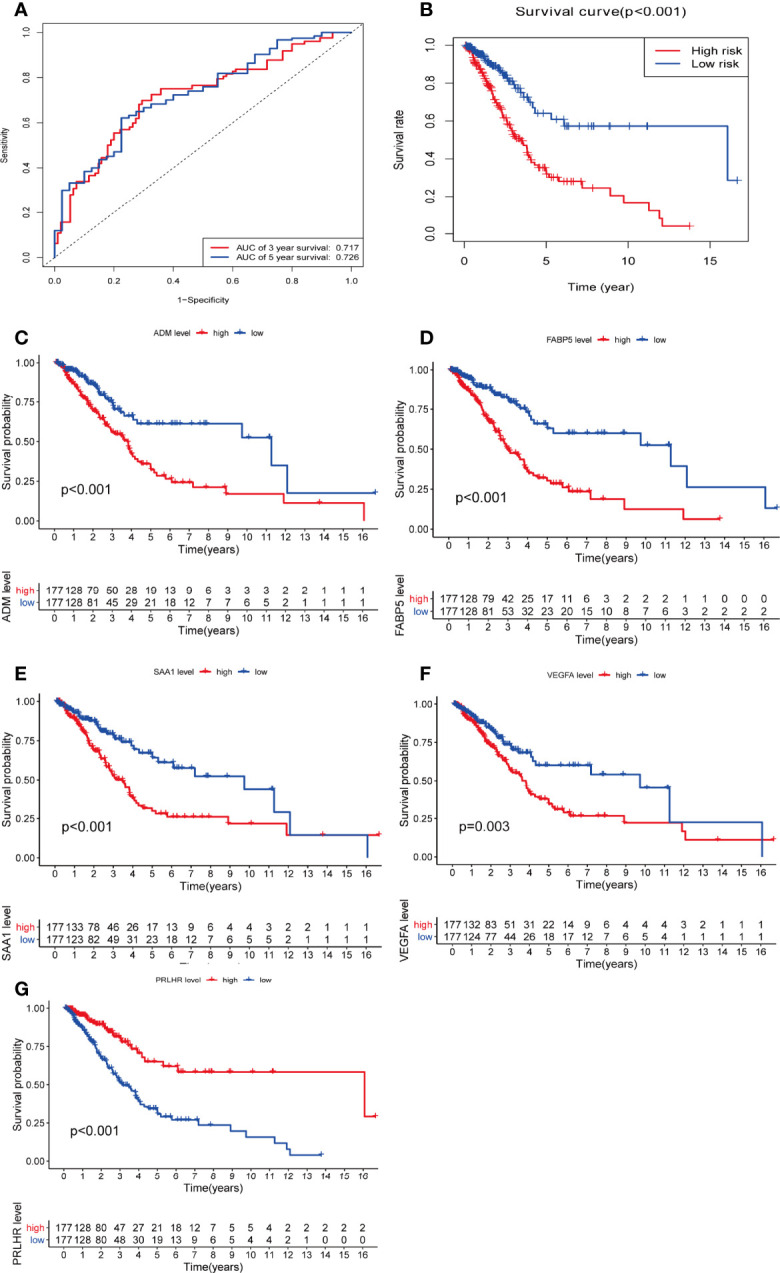
There is a significant difference in overall survival rate among glioma patients when assessed with the five DEmRNAs. **(A)** Time-dependent receiver operating character curve of the prognosis model constructed using 5-DEmRNA. **(B)** Overall survival rate of two groups of patients. **(C–G)** Kaplan–Meier analysis estimates of the overall survival rate using the 5-DEmRNAs in both groups of patients. red, high risk; blue, low risk.

Additionally, we used time-dependent ROC analysis to assess the prognostic significance of the five DEmRNAs. ROC analysis indicated that the system showed promising prognostic prediction (3-year AUC = 0.717, 5-year AUC = 0.726) (**Figure 5A**).

Based on risk score, 354 patients with glioma were divided into low and high risk groups. There was a significant difference in OS between the two groups. The higher the risk score, the shorter the OS time and the higher the mortality (p < 0.05, **Figure 5B**). The relationship between the five DEmRNAs and OS was analyzed in the two groups (P < 0.05). Significant differences in OS rate were observed between the low and high expression groups. *FABP5, VEGFA, SAA1*, and *ADM* survival analysis showed that the higher the risk score, the higher the risk of death. *PRLHR* Kaplan-Meier analysis showed that the lower the risk score, the higher the risk of death (P < 0.05) (**Figures 5C–G**). These results suggested that *PRLHR* may inhibit tumor progression in gliomas.

### Analysis of the Clinical Correlation Between Five Prognostic Related Immune Genes and Glioma

**Figure 6:**
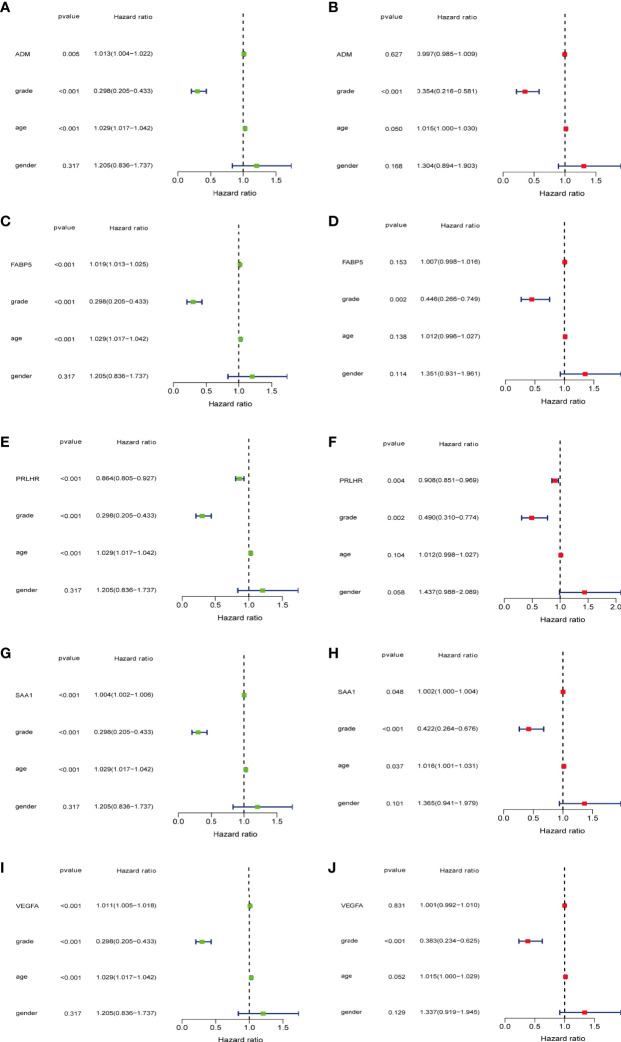
5-DEmRNAs are analyzed separately with age, gender, and tumor grade for independent prognostic analysis. **(A, C, E, G, I)** Single factor Cox regression analysis. **(B, D, F, H, J)** Multivariate Cox regression analysis. *PRLHR* can be used as an independent prognostic factor for patients with glioma.

The expression values of five prognosis-related immune genes were integrated with clinical information (age, gender, and tumor grade)(**Supplementary Data 2**). Univariate and multivariate independent prognostic analysis showed that *PRLHR* and *SAA1* were the independent predictor of glioma (univariate and multivariate analysis P < 0.05, **Figures 6A–J**). *PRLHR* expression is negatively correlated with survival. PRLHR may be a tumor suppressor in gliomas. So we selected *PRLHR* as the focus of our next research.

**Figure 7:**
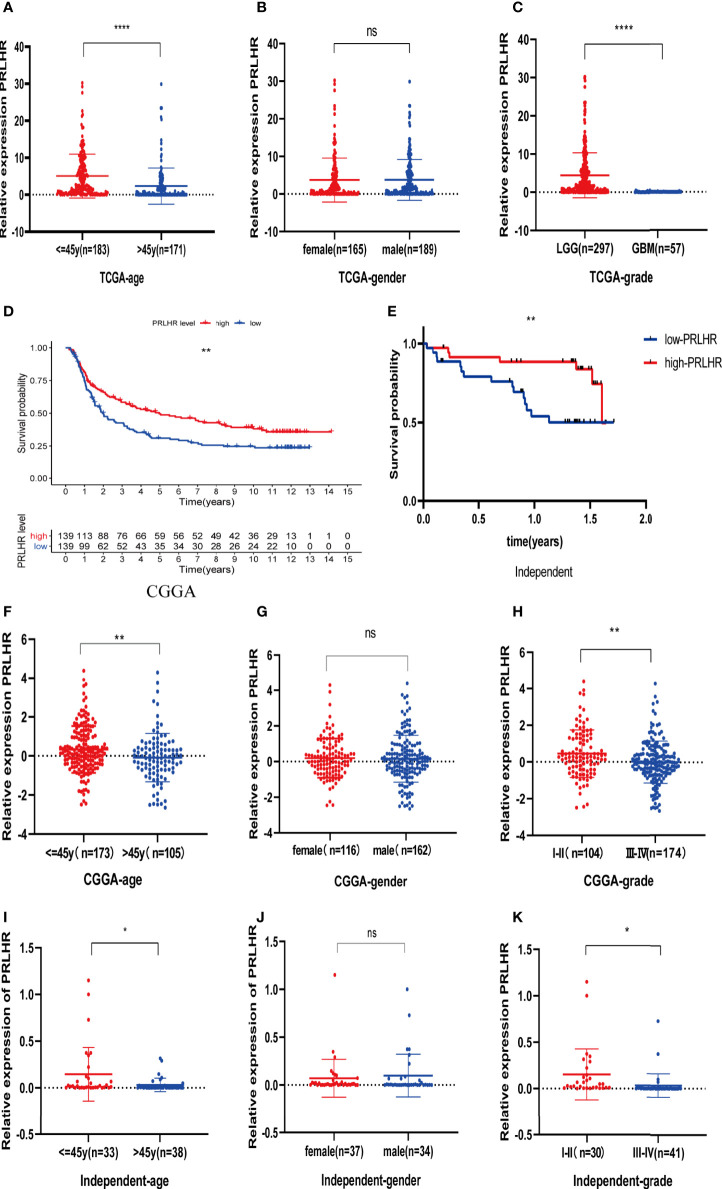
Clinical correlation (age, gender and grade) analysis of *PRLHR* in TCGA, CGGA, and independent data. **(A–C)** Analysis of clinical correlation of *PRLHR* in TCGA **(D, E)**. *PRLHR* expression is related to OS of patients with glioma in CGGA and independent data **(F–K)**. Clinical correlation analysis of *PRLHR* in CGGA and independent data. *p < 0.05, **p < 0.01, ****p < 0.0001, ns (not significant) p > 0.05.

Our results showed that *PRLHR* expression is lower in patients aged > 45 years than in those aged ≤ 45 years, suggesting that prognosis may be worse in patients older than 45 years. There is no difference in *PRLHR* with gender. *PRLHR* expression in patients with LGG is higher than that in patients with GBM. This suggests that the prognosis of GBM is worse, which is consistent with clinical observation. Taken together, these results show that *PRLHR* can be used as a marker to predict the prognosis of patients with glioma (**Figures 7A–C**).

### CGGA and Independent Sample Verification

Survival analysis showed that *PRLHR* expression was negatively correlated with survival (**Figures 7D, E**), which was verified by CGGA data and independent sample data (**Table 2**). Clinical correlation analysis of *PRLHR* expression showed reduced *PRLHR* expression in the > 45 years age group, suggesting that prognosis of the patients in this age group may be worse (**Figures 7F, I**). There was no difference in the expression of *PRLHR* in gender groups (**Figures 7G, J**). Expression of *PRLHR* in patients with low-grade gliomas (WHO I-II) is higher than that in patients with high-grade gliomas (WHO III-IV), including GBM, suggesting that the prognosis of patients with high-grade gliomas is worse (**Figures 7H, K** and **Supplementary Data 3, 4**). It can be concluded that *PRLHR* can be used as a marker to predict the prognosis of patients with glioma.

### GO and KEGG

**Figure 8:**
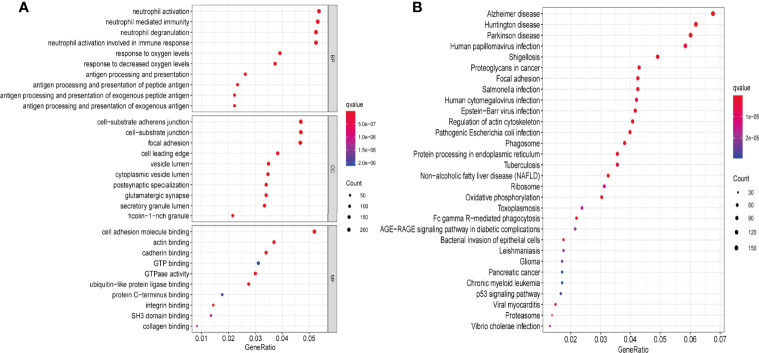
PRLHR functional enrichment analysis. **(A)** The function of PRLHR in GO (biological process [BP], cellular component [CC], and molecular function [MF]) enrichment (top 10, p < 0.05); **(B)** KEGG pathway enrichment (top 30, p < 0.05).

The transcriptome data of 698 glioma tissues was downloaded from TCGA database and divided into two groups based on PRLHR expression. After screening using the limma package, 5145 DEGs were identified(|log2FC|>2 and P < 0.001). GO enrichment and KEGG pathway analyses were performed on these 5145 DEGs. GO enrichment analysis results show that these DEGs are mainly involved in antigen processing and foreign antigen presentation, antigen processing and presentation, antigen processing and peptide antigen presentation, antigen processing and exogenous peptide antigen presentation, neutrophil mediated immunity, neutrophil activation involved in immune response, neutrophil degranulation, and other immune-related functions (**Figure 8A**). In addition, KEGG pathway analysis included glioma-related signal pathway, and tumor-related p53 signal pathway (**Figure 8B**).

## Discussion

Glioma is a malignant tumor characterized by multiple immune cell infiltration and complicated immune response. Identification of prognosis-related immune genes to benefit patients with glioma is important as the prognosis of these patients remains poor. Traditionally, it was thought that the blood-brain barrier prevented immunotherapy from working in the central nervous system. However, more recent reports suggest that immunotherapy may be beneficial in the treatment of central nervous system cancers (20). Immunotherapy, including vaccine therapy, checkpoint inhibitors, chimeric antigen TCR, and viral immunotherapy, can improve the prognosis and OS in patients with gliomas (21). Immune regulation between immune cells or between tumor cells and immune cells promotes tumor progression (22). Therefore, it is important to identify immune target genes related to the prognosis to improve the survival rate of patients with gliomas.

TMB is an important biological marker that reflects the degree of tumor mutation. Alexandrov and Lawrence et al. found significant differences in TMB among different tumor samples, ranging from 0.001–400/Mb. There were also significant differences in TMB among different patients with the same tumor type. TMB has been reported as a biomarker that correlates with tumor immunotherapy efficacy (23). The reason that TMB has become a marker of immunotherapy stems from the biological mechanism of somatic mutation and immune response. Somatic mutations in tumors include synonymous mutations and non-synonymous mutations. Non-synonymous mutations produce abnormal proteins by changing the amino acid sequence. The immunogenicity of abnormal proteins in tumors is the basis of the tumor immune response. If abnormal proteins are eventually recognized by immune cells, they will become new antigens, and the immune response can subsequently develop (24). Therefore, when the TMB of the tumor sample is high, mutations that produce a new immunogenic antigens in the tumor are also increased. This makes it easier for the immune system to identify and clear tumor cells, and the patient survival rate will be relatively improved. Consistent with this, the OS of glioma patients in the high TMB group was significantly higher than that in the low TMB group in our study. In addition, we also identified a statistical correlation between TMB and glioma grade and age.

We obtained 2325 DEGs, related to TMB, and performed GO enrichment and KEGG pathway analyses. The results showed that these DEGs were mainly involved in immune-related functions, including humoral immune response, leukocyte migration, and circulating immunoglobulin-mediated humoral immune response. Based on univariate and multivariate cox regression analysis, we identified five genes, *FABP5, VEGFA, SAA1, ADM*, and *PRLHR*, for prognosis prediction in patients with glioma. ROC curve analysis showed that this model is reliable in predicting the prognosis of patients with glioma. However, more clinical trials are required to verify these results.

Immune cells may play different roles in the development of tumors (25). In our study, we calculated the proportion of 22 different immune cells in gliomas. Patients with glioma were divided into two groups based on their TMB score. The infiltration levels of CD8+T cells, helper T cells, γ δ T cells (T cells gamma delta), M0, M1, M2, and neutrophils were higher in the high TMB group than in the low TMB group. Meanwhile, the proportion of activated NK cells, monocytes, and activated mast cells were higher in the low TMB group than in the high TMB group. Recent studies reported that CD8+T cells, helper T cells, γ δ T cells, and cytoskeletal M2 cells play important roles in anti-tumor immunity (26–28). We speculate that high TMB can induce anti-tumor activation of immune cells and improve the prognosis of glioma patients.

PRLHR (prolactin releasing hormone receptor), or G protein coupled receptor 10, is the receptor for prolactin releasing peptide (PrRP). *PRLHR* is associated with feeding and the regulation of energy balance (29). Additionally, *PRLHR* mutation is a protective factor in Chinese Han patients with colorectal cancer (30). Some studies have shown a negative correlation between the *PRLHR* expression levels and immune cells infiltration in LGG, but the role of *PRHLR* in glioma remains unclear (31). We integrated the expression of five prognosis-related immune genes with clinical information (age, sex, and tumor grade). Univariate and multivariate independent prognostic analysis showed that *PRLHR* was the only independent predictor of glioma. Our results show that *PRLHR* expression is negatively correlated with survival, and that the survival rate of patients with low *PRLHR* expression is decreased. Therefore, PRLHR may be a suppressor of glioma tumorigenesis. We also found that *PRLHR* expression is lower in patients in the > 45 year age group than that in patients in the ≤ 45 year age group, suggesting that the prognosis of patients with over 45 years of age may be worse. *PRLHR* expression in patients with low-grade gliomas is higher than that in patients with high-grade gliomas, including GBM, suggesting that the prognosis of high-grade gliomas is worse. There was no observed difference between gender and high-grade gliomas. Some studies have shown that the grade of glioma increases with increased age, and the survival rate of I~IV grade glioma decreases with the increased grade (32). Therefore, *PRLHR* may be a protective factor for the occurrence of gliomas and can be used as an independent prognostic marker for patients with gliomas.

We used GO and KEGG enrichment analyses to predict the role of *PRLHR* in glioma. The results show that *PRLHR* may inhibit the occurrence and development of glioma by regulating immune cells and their ability to participate in the immune response. However, further verification of cellular function is still needed.

## Conclusion

*PRLHR* is an immune gene related to TMB, and significantly differs from tumor grade and age of patients with glioma. Moreover, *PRLHR* is related to the prognosis and survival rate of patients with glioma. *PRLHR* can be used as a tumor inhibitory factor for the development of glioma, and has important guiding significance for the prognosis and immunotherapy of glioma.

## Data Availability Statement

The datasets presented in this study can be found in online repositories. The names of the repository/repositories and accession number(s) can be found in the article/**Supplementary Material**.

## Ethics Statement

The studies involving human participants were reviewed and approved by Ethics Committee of Xiangya Hospital of Central South University. The patients/participants provided their written informed consent to participate in this study.

## Author Contributions

YL designed the experiment, collected and analyzed the data, YL and JX wrote the manuscript, and CF S and GP checked the manuscript. All authors contributed to the article and approved the submitted version.

## Funding

This study was supported by the National Natural Science Foundation of China Youth Fund Project (no. 81801908), Hunan Provincial Natural Science Committee Project (no. 2021JJ70074) and Hunan Province Science and Technology Major Project(no.2019SK1010).

## Conflict of Interest

The authors declare that the research was conducted in the absence of any commercial or financial relationships that could be construed as a potential conflict of interest.

## Publisher’s Note

All claims expressed in this article are solely those of the authors and do not necessarily represent those of their affiliated organizations, or those of the publisher, the editors and the reviewers. Any product that may be evaluated in this article, or claim that may be made by its manufacturer, is not guaranteed or endorsed by the publisher.
